# Operation for Hare-lip, &c

**Published:** 1866-06

**Authors:** E. Daniels

**Affiliations:** Owego


### OPERATION FOR HARE-LIP, &c.

*Messrs. Editors,*—In a recent number of your *Journal* I observe a suggestion in reference to an improvement in the construction of hare lip pins, from Dr. S. B. S., of Exeter, N. H. I have no doubt of the utility of what the Doctor suggests; but at the same time I think my own method of operating preferable.

I have had occasion, within the last ten years, to operate for hare lip a very considerable number of times, and have invariably used the finest sewing needles that I could obtain. I insert them with a suitable pair of pliers (a great deal better, by the way, than the fingers for this purpose), and take care to make the insertion deep and thorough. After the parts are securely bound with sutures, I cut off both ends of the needles and cover them with adhesive plaster. The children appear to enjoy themselves at the breast as well as before the operation. In every instance in my experience thus far, the succes has been perfect, and my conviction is, that every surgeon who gives this method a fair trial will never after use anything else. I usually insert three needles.

I have also practised the dressing of other incised wounds in the same way, with very satisfactory results. Only yesterday after the amputation of the mamma, I dressed in this way, because from previous experience I am satisfied of its superiority over the ordinary suture. In securing lacerated and incised injuries of the scalp, there is no comparison between the use of these fine needles and any others I ever saw used. In most cases it is entirely unnecessary to remove any of the hair; and the parts tolerate the presence of the needles much better than they do that of any suture.

*Owego, April 4th* 1865. Very truly, &c.,

E. Daniels, M. D.

[The above method of operating for hare-lip is essentially that recommended by Prof. Gross, in his great work on Surgery, as the best. Dr. Gross, however, does not find it necessary to use any plasters.—Eds.]

